# Hypoxia represses FOXF1 in lung endothelial cells through HIF-1α

**DOI:** 10.3389/fphys.2023.1309155

**Published:** 2024-01-11

**Authors:** Anusha Acharya, Fenghua Bian, Jose Gomez-Arroyo, Kimberly A. Wagner, Vladimir V. Kalinichenko, Tanya V. Kalin

**Affiliations:** ^1^ Division of Pulmonary Biology, Cincinnati Children’s Hospital Medical Center, Cincinnati, OH, United States; ^2^ Division of Pulmonary and Critical Care and Sleep Medicine, Department of Internal Medicine, University of Cincinnati, Cincinnati, OH, United States; ^3^ Phoenix Children’s Health Research Institute, University of Arizona College of Medicine—Phoenix, Phoenix, AZ, United States; ^4^ Division of Neonatology, Phoenix Children’s Hospital, Phoenix, AZ, United States

**Keywords:** lung, endothelial cells, hypoxia, FOXF1, HIF-1α

## Abstract

**Introduction:** Forkhead Box F1 (FOXF1) transcription factor plays a critical role in lung angiogenesis during embryonic development and lung repair after injury. FOXF1 expression is decreased in endothelial cells after lung injury; however, molecular mechanisms responsible for the FOXF1 transcript changes in injured lung endothelium remain unknown.

**Methods:** We used immunostaining of injured mouse lung tissues, FACS-sorted lung endothelial cells from hypoxia-treated mice, and data from patients diagnosed with hypoxemic respiratory failure to demonstrate that hypoxia is associated with decreased FOXF1 expression. Endothelial cell cultures were used to induce hypoxia *in vitro* and identify the upstream molecular mechanism through which hypoxia inhibits FOXF1 gene expression.

**Results:** Bleomycin-induced lung injury induced hypoxia in the mouse lung tissue which was associated with decreased *Foxf1* expression. Human *FOXF1* mRNA was decreased in the lungs of patients diagnosed with hypoxemic respiratory failure. Mice exposed to hypoxia exhibited reduced *Foxf1* expression in the lung tissue and FACS-sorted lung endothelial cells. *In vitro*, hypoxia (1% of O_2_) or treatment with cobalt (II) chloride increased HIF-1α protein levels but inhibited *FOXF1* expression in three endothelial cell lines. Overexpression of HIF-1α in cultured endothelial cells was sufficient to inhibit *Foxf1* expression. siRNA-mediated depletion of HIF-1α prevented the downregulation of *Foxf1* gene expression after hypoxia or cobalt (II) chloride treatment.

**Conclusion:** Hypoxia inhibits FOXF1 expression in endothelial cells in a HIF-1α dependent manner. Our data suggest that endothelial cell-specific inhibition of HIF-1α via gene therapy can be considered to restore FOXF1 and improve lung repair in patients with severe lung injury.

## Introduction

The lung is a well-oxygenated organ; however, multiple pathological conditions exist that result in the tissue becoming hypoxic (Shimoda and Semenza, 2011). These include acute respiratory distress syndrome (ARDS), acute lung injury (ALI), COVID-19 and influenza viral infections, bacterial pneumonia, chronic obstructive pulmonary disease (COPD), idiopathic pulmonary fibrosis (IPF), cystic fibrosis, bronchopulmonary dysplasia and lung cancer (Carbone et al., 2005; Naeije, 2005; Stenmark and Abman, 2005; Strange and Highland, 2005; Wigley et al., 2005; Ball et al., 2014). Additionally, hypoxia frequently correlates with disease severity and it is associated with poor outcomes in patients with IPF and COPD (Tzouvelekis et al., 2007; Bodempudi et al., 2014; Weng et al., 2014; Aquino-Gálvez et al., 2019).

The transcription factor Forkhead Box F1 (FOXF1) is an evolutionarily conserved member of the Forkhead Box (FOX) family of transcription factors. During embryonic development, FOXF1 functions as an essential mediator of angiogenesis (Mahlapuu et al., 2001a; Mahlapuu et al., 2001b; Claxton et al., 2008; Ren et al., 2014). Heterozygous deletions and loss-of-function point mutations in the *FOXF1* gene locus are associated with alveolar capillary dysplasia with misalignment of pulmonary veins (ACDMPV). ACDMPV is a rare congenital disease occurring in neonates and infants which is characterized by impaired development of the alveolar capillary network, misalignment of pulmonary veins, and severe pulmonary hypertension (Bishop et al., 2011). Global deletion of *Foxf1* (*Foxf1* ^*−/−*^) in mice is embryonically lethal, whereas mice with heterozygous deletion of *Foxf1* (*Foxf1* ^*+/−*^) present with alveolar capillary dysplasia along with developmental defects in the lung, intestine, and liver (Mahlapuu et al., 2001a; Mahlapuu et al., 2001b; Kalinichenko et al., 2002a; Kalinichenko et al., 2002b; Kalinichenko et al., 2003; Claxton et al., 2008). Conditional deletion of *Foxf1* specifically in endothelial cells results in embryonic lethality due to impaired VEGF signaling in the yolk sac, placenta, and lung (Ren et al., 2014). FOXF1 stimulates the expression of genes critical for endothelial barrier function and plays an important role in maintaining normal lung homeostasis and lung repair after injury (Cai et al., 2016).

We previously showed that pulmonary endothelial cells isolated from bleomycin-treated mice and human IPF lungs exhibited low levels of *FOXF1* (Bian et al., 2023). Further, employing a transgenic mouse model with endothelial-specific deficiency of *Foxf1*, we demonstrated that loss of *Foxf1* exacerbated the fibrotic phenotype. FOXF1 has previously been reported as an anti-fibrotic factor preventing the reprogramming of lung fibroblasts into myofibroblasts in mouse and human lungs (Black et al., 2018). While the FOXF1 expression is decreased after lung injury, molecular mechanisms through which *FOXF1* gene expression changes occur remain unknown.

In our previous studies, the downregulation of FOXF1 in endothelial cells corresponded with diminished arterial oxygenation and hypoxemia in mouse lung tissue (Cai et al., 2016; Pradhan et al., 2019; Ren et al., 2019; Bolte et al., 2020; Wang et al., 2021; Wang et al., 2022). However, the direct impact of hypoxia on the expression of FOXF1 in pulmonary endothelial cells is unclear.

In the present study, we aimed to determine if the hypoxic microenvironment plays a role in the downregulation of FOXF1 in injured lung endothelial cells *in vitro* and *in vivo*. We showed that hypoxia inhibits FOXF1 in endothelial cells in a HIF-1α dependent manner. Our data suggest that specific inhibition of HIF-1α in endothelial cells via gene therapy can be considered to increase FOXF1 and improve lung repair in patients with severe lung injury.

## Materials and methods

### Bleomycin-induced lung injury

To induce pulmonary fibrosis, mice were administered 2 or 2.5 U/kg of bleomycin sulfate (EMD Biosciences) intratracheally (I.T.) once. The control group of mice (Uninjured) was administered PBS I.T. the same way as bleomycin.

### Detection of hypoxia in bleomycin model of lung injury

3 days post-induction of bleomycin injury, hypoxia was assessed in the mouse lungs as previously described (Cantelmo et al., 2016). Briefly, mice were injected with 60 mg/kg pimonidazole hydrochloride (Hypoxyprobe™ kit, cat#HP1) 1 hour prior to sacrifice. To detect the formation of pimonidazole adducts, paraffin sections were immunostained with hypoxyprobe-1-mAb1 following the manufacturer’s instructions.

### *In vivo* model of hypoxia

Wild-type C57BL/6 mice were purchased from the Jackson Laboratory. Mice were housed in specific pathogen-free animal facilities at the Cincinnati Children’s Hospital Medical Center. Experiments were conducted on 8–12 weeks old age-matched mice. For hypoxia exposure, mice were housed inside a nitrogen dilution hypoxia chamber set to a fraction of inspired oxygen of 10%. The oxygen concentration was monitored and controlled with a ProOx model 350 unit (BioSpherix) by infusion of Nitrogen. In parallel, a separate set of wild-type mice were kept in normal room air (Normoxia). On day 3, day 7 and day 10 of hypoxia exposure, mice were euthanized, and the lungs were perfused with PBS and harvested. All methods and protocols were approved by the Animal Care and Use Committee of the Cincinnati Children’s Hospital Medical Center.

### Cell lines and reagents

Human endothelial HUVEC cells (Lonza, Cat# C2519A) were cultured in EGM2/EBM2 (Lonza) growth medium, and Human Pulmonary Artery Endothelial Cells (HPAEC, Cat# CC2530) were cultured in ECM Medium (ScienCell). HUVEC and HPAEC cells were from pooled donors. Mouse endothelial MFLM-91U cells (Seven Hills, Cat# AMFLM-91U) were cultured in DMEM (Gibco).

### *In vitro* models of hypoxia

For hypoxia exposure, cells were grown to 70% confluency and were changed to fresh complete media before hypoxia treatment using a finely controlled Whitley H35 Hypoxystation (Don Whitley Scientific). The oxygen concentration in the chamber was maintained at 1% with a residual gas mixture composed of 5% carbon dioxide and balanced nitrogen. Normoxia-treated cells used as a control were cultured in 95% atmospheric air and 5% carbon dioxide and 21% oxygen concentration. For a chemically induced model of hypoxia, cells were grown to 70% confluency and changed to fresh complete media with a final concentration of 150 µM of cobalt (II) chloride (Sigma Aldrich, Cat# 60818).

### Flow cytometry

Flow cytometry was performed on single cell suspensions from enzyme-digested whole lungs as described previously (Bian et al., 2023). Briefly, whole lungs were dissociated with Liberase, washed with PBS, filtered through a 70-um filter and counted to get the final cell count of the single cell suspension. Live cells were detected with 7-amino actinomycin D (7-AAD) (BioLegend). Hematopoietic cells were detected using CD45 antibody (Biolegend, Cat# 103140). The CD45^−^ population was then assessed for expression of CD31 (eBioscience, Cat# 48-0311-82). Endothelial cells that were identified as CD31^+^ and CD45^−^ were FACS-sorted using the FACSAria II or FACSymphony S6, five-laser cell sorter (BD Biosciences). After FACS-sorting, the CD45^−^CD31^+^ endothelial cell population is >95% pure and lacks fibroblasts and other mesenchymal and epithelial cells as previously described (Ren et al., 2014; Bolte et al., 2017; Sun et al., 2021; Wen et al., 2021; Kolesnichenko et al., 2023).

### qRT- PCR and Western blot

For the knockdown of *Hif1α*, MFLM-91U cells were transfected with si*Hif1α* (Horizon Discovery, Cat#M-040638-00-0010) using Dharmafect transfection reagent 1 according to the manufacturer’s protocol (Dharmacon). A cocktail of non-targeting siRNAs was used as a control (Horizon, Cat# D-001810-01-20). For overexpression of HIF-1α, cells were transfected with CMV-HIF-1α (Origene, Cat# MG210895). Cells were lysed for RNA and protein using lysis buffer (Qiagen, cat#74104) and RIPA buffer (Cell Signaling Technology, Cat#9806) respectively, as per the manufacturer’s protocol. qRT-PCR was performed as previously described (Kalin et al., 2008) using TaqMan probes for mouse *Foxf1* (Mm00487497_m1), mouse *Hif1α* (Mm00468869_m1), mouse *ActB* (Mm00607939_s1), human *FOXF1* (Hs00230962_m1), human *HIF1α* (Hs00153153_m1) and human *ACTB* (Hs99999903_m1). Protein extracts were prepared as described (Bolte et al., 2015; Milewski et al., 2017). The following antibodies were used for western blots: anti-HIF-1α (Novus Biologicals, Cat# NB100-449), anti-FOXF1 (RnD systems, Cat#AF4798) and anti-Beta Actin (Santacruz, Cat# sc47778).

### Human RNA seq

Human samples analyzed were obtained as part of a larger dataset deposited from the Pulmonary Hypertension Breakthrough Initiative (PHBI) Biobank (Stearman et al., 2018). All samples utilized in this study have been previously characterized, processed and de-identified accordingly, as part of the PHBI Biobank. We have obtained permission to use samples, as well as clinical data for research and publication as per the most recent MTA. Patient enrolment, tissue-processing protocol, RNA isolation and library preparation for all PHBI samples have been previously described (Stacher et al., 2012; Stearman et al., 2018). Only datasets from controls were used for analysis. Paired-end 75 base-pair RNA sequencing (RNA-seq) was performed on all available lung samples using an Illumina sequencer. Samples were sequenced in two batches. The sequencing depth was 20–25 million reads per sample in one batch and 15–20 million reads per sample in the other batch. Reads were pseudo-aligned and quantified using an index transcriptome version of the GRCh38.p14 human genome (RefSeq GCF_000001405.40) using *Kallisto* with standard settings (Bray et al., 2016). Raw counts were normalized using *DESeq2* (Love et al., 2014). Normalized counts were compared between groups using non-parametric testing with GraphPad PRISM v.10.

### Statistical analysis

The Student t-test (two-tailed), non-parametric Mann-Whitney U test and one-way ANOVA followed by Dunnett’s multiple comparison test were used to determine statistical significance. *p*-values < 0.05 were considered significant. Values were shown as mean ± standard deviation (SD). All statistical analyses were obtained using GraphPad PRISM v.10 for Windows.

### Study approval

All animal studies were approved by the Cincinnati Children’s Research Foundation Institutional Animal Care and Use Committee and covered under our animal protocol (IACUC 2022-0041). The Cincinnati Children’s Research Foundation Institutional Animal Care and Use Committee is an AAALAC and NIH accredited institution (NIH Insurance #8310801).

## Results

### Hypoxia is associated with decreased *FOXF1* expression in injured mouse and human lungs

**FIGURE 1:**
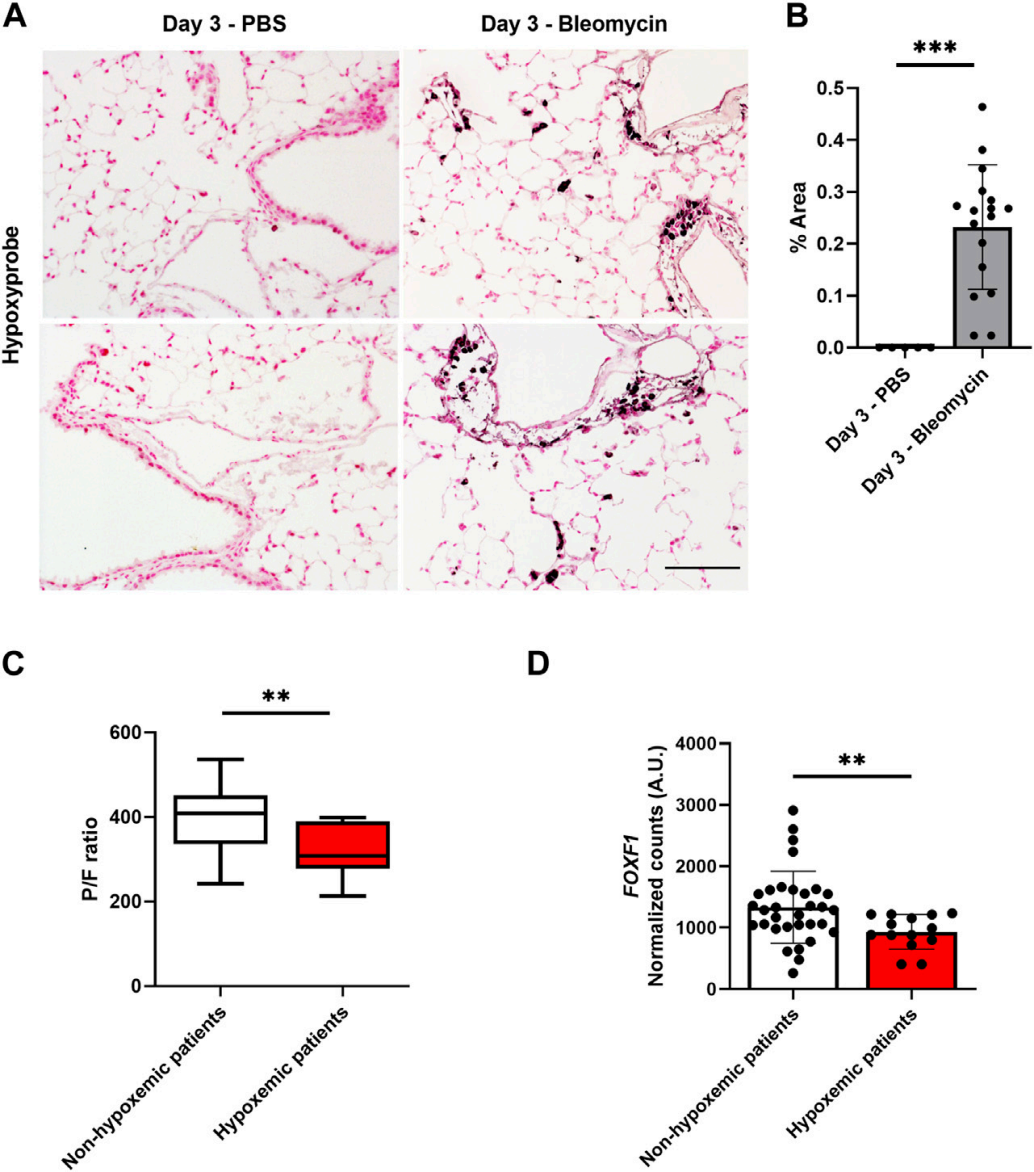
Hypoxia is associated with decreased *Foxf1* expression in injured mouse and human lungs. **(A,B)** Immunohistochemical staining of mouse lung sections for hypoxyprobe-1-mAb1 shows bleomycin-injured lungs have increased hypoxia at day 3 post-injury induction. Hypoxyprobe area was calculated as % of hypoxyprobe-1-mAb1 positive area per field in 5 random fields using NIS elements software version 4.5. Each dot represents a single field. N = 3 mice per group. Both male and female mice were included. Scale bar = 50 µm. Values are shown as mean ± SD. *, *p* < 0.05; **, *p* < 0.01; ***, *p* < 0.001 from Student’s *t* test (two-tailed). **(C)** Hypoxemic patients had a P/F value of less than 400. The P/F ratio of patients was calculated as the partial pressure of oxygen in blood divided by the ratio of the inspired fraction of oxygen (P/F ratio) provided by the mechanical ventilator. N = total 46 patients. Values are shown as mean ± SD. *, *p* < 0.05; **, *p* < 0.01; ***, *p* < 0.001 from non-parametric Mann-Whitney U test. **(D)** Hypoxemic patients have decreased *FOXF1* expression compared to non-hypoxemic patients. Normalized counts of *FOXF1* expression were obtained from the RNA seq data set. N = total 46 patients. Each dot represents a patient. Values are shown as mean ± SD. *, *p* < 0.05; **, *p* < 0.01; ***, *p* < 0.001 from non-parametric Mann-Whitney U test.

Our published studies demonstrated that bleomycin-induced lung injury decreases the expression of *Foxf1* in pulmonary endothelial cells in a time-dependent manner (Bian et al., 2023). This decrease was observed at a time point as early as 3 days after the bleomycin treatment (Bian et al., 2023). Since hypoxia is frequently associated with lung injury, we tested if hypoxia was induced in bleomycin-injured mouse lungs. To determine whether the lung injury affected the oxygen availability in the lung tissue 3 days after the bleomycin treatment, we injected control (PBS injected) and bleomycin-injured mice with pimonidazole, a chemical sensor for hypoxia (Cantelmo et al., 2016). Bleomycin injury increased the hypoxic area in the lung tissue compared to control lungs (Figures 1A, B). Thus, the hypoxia in bleomycin-injured mouse lungs directly correlates with reduced *Foxf1* expression shown previously (Bian et al., 2023).

Next, we examined if hypoxia is associated with decreased *FOXF1* expression in human lungs. Explanted lung tissue from failed donor patients was evaluated. Briefly, these patients became unsuitable to donate after tissue procurement for various reasons including hypoxemic respiratory failure, ABO blood type incompatibility, hemodynamic instability, etc., First, we separated patients by their capacity to oxygenate blood directly assessed by the partial pressure of oxygen in blood divided by the ratio of the inspired fraction of oxygen (P/F ratio) provided by the mechanical ventilator (Figure 1C). P/F values below 400 indicate hypoxemia. This ratio is clinically used to assess for and classify the severity of hypoxemic respiratory failure (Ranieri et al., 2012). Using this dataset, we then analyzed *FOXF1* expression in the patients who were hypoxemic before transplantation compared to non-hypoxemic patients. The *FOXF1* expression in hypoxemic patients was significantly decreased compared to the patients without hypoxemia (Figure 1D). Thus, the correlation between hypoxemia and decreased *FOXF1* expression in human and mouse lungs prompted us to investigate whether *FOXF1* expression is regulated by oxygen levels.

### Hypoxia inhibits *Foxf1* in lung endothelial cells *in vivo*

**FIGURE 2:**
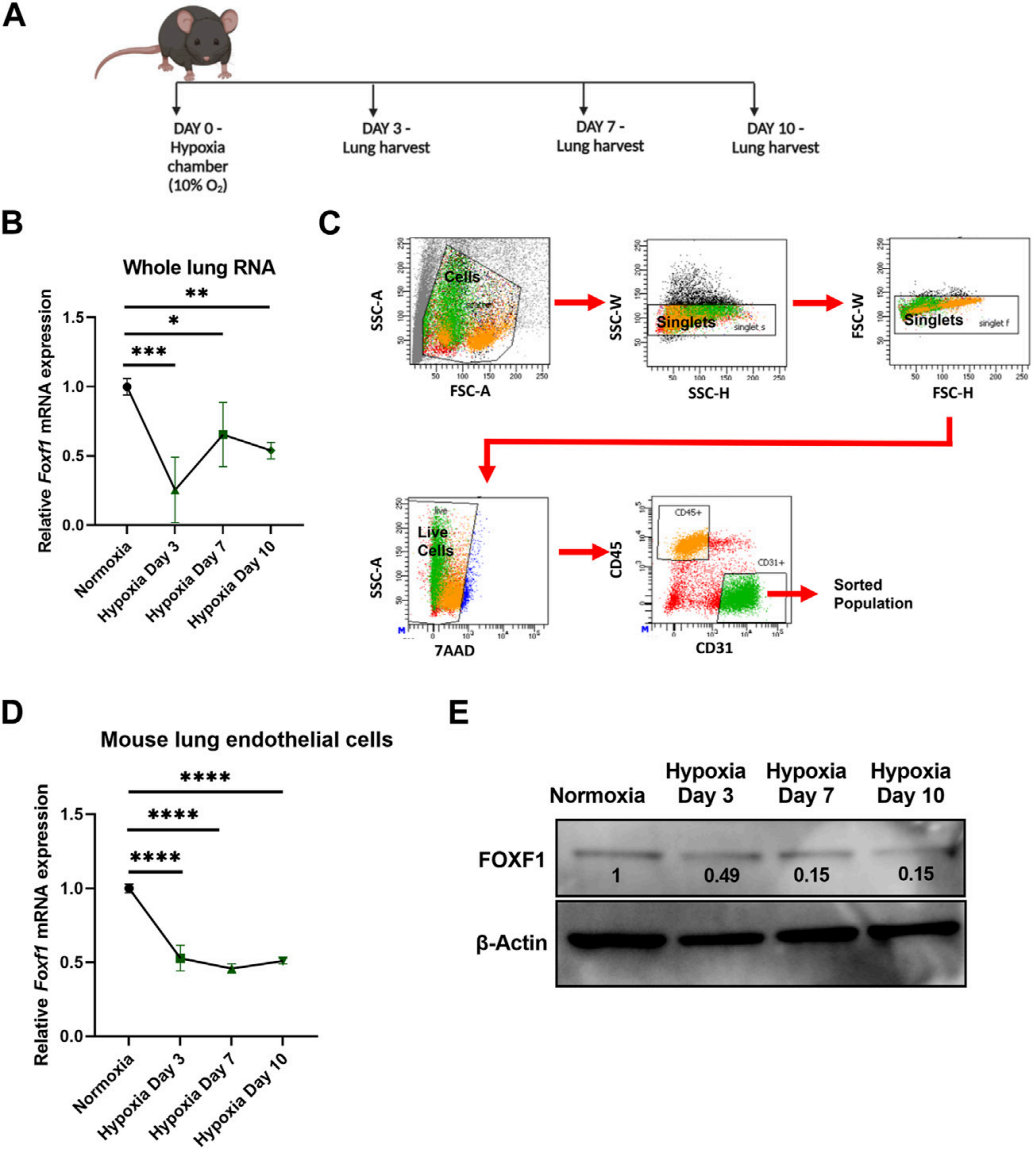
Hypoxia inhibits *Foxf1* in lung endothelial cells *in vivo* **(A)** Experimental schematic showing timeline of exposure of 8–12 weeks old Wild-type C57BL/6 mice to hypoxia or normoxia and lung harvest. **(B)** *Foxf1* mRNA expression was decreased in mouse lungs at day 3, day 7 and day 10 after exposure to hypoxia. qRT-PCR was performed on total RNA isolated from harvested lung tissue. β-Actin (*Actb*) was used as the housekeeping gene. N = 4 mice per group. **(C)** The gating strategy utilized to FACS sort CD45^-^/CD31^+^ endothelial cells from mouse lungs exposed to hypoxia or normoxia. **(D)** *Foxf1* mRNA expression was decreased in CD45^−^/CD31^+^ endothelial cells FACS sorted from mouse lungs at day 3, day 7 and day 10 post hypoxic exposure. qRT-PCR analysis was performed in triplicate on pooled RNA samples from each group. β-Actin (*Actb*) was used as the housekeeping gene. N = 4 mice per group. **(E)** FOXF1 protein expression was decreased in CD45^-^/CD31^+^ endothelial cells FACS sorted from mouse lungs at day 3, day 7 and day 10 post hypoxic exposure. Values on the western blot image represent FOXF1 protein expression relative to normoxic condition. Densitometric analysis was done using ImageJ software. Values were normalized to housekeeping protein β-Actin. Western blot was performed on pooled protein samples from each group. N = 4 mice per group. All values in this figure are shown as mean ± SD. *, *p* < 0.05; **, *p* < 0.01; ***, *p* < 0.001 from one-way ANOVA followed by Dunnett’s test.

Since downregulation of *Foxf1* expression coincided with hypoxia in bleomycin-injured mouse lungs, we examined if hypoxia alone is sufficient to decrease the *Foxf1* expression in pulmonary endothelial cells. To investigate this possibility, we exposed age-matched adult wild-type C57BL/6 mice to continuous hypoxia (10% oxygen) or normoxia (21% oxygen) for 3, 7, and 10 days (Figure 2A). *Foxf1* mRNA was measured in whole lung RNA obtained from mice exposed to either hypoxia or normoxia (control). *Foxf1* mRNA was decreased in the lungs of hypoxic mice as early as day 3 and remained decreased at day 7 and day 10 post hypoxic exposure (Figure 2B). Since in the adult lung expression of *Foxf1* is the highest in endothelial cells (Kalucka et al., 2020; Paik et al., 2020), we measured *Foxf1* mRNA in purified lung endothelial cells obtained from hypoxic and normoxic mice. FACS sorting of CD31^+^/CD45^−^ lung endothelial cells was performed at different time points after hypoxic and normoxic exposure (Figure 2C). Endothelial *Foxf1* mRNA was measured by qRT-PCR. Similar to the total lung RNA data (Figure 2B), *Foxf1* mRNA in FACS-sorted lung endothelial cells was reduced starting at day 3 after the initiation of hypoxia exposure (Figure 2D). Western blot analysis was consistent with reduced FOXF1 protein amounts in the lung tissue after hypoxia (Figure 2E). Thus, hypoxia inhibits *Foxf1* expression in mouse lung endothelial cells *in vivo*.

### Hypoxia inhibits *Foxf1* in lung endothelial cells *in vitro*

**FIGURE 3:**
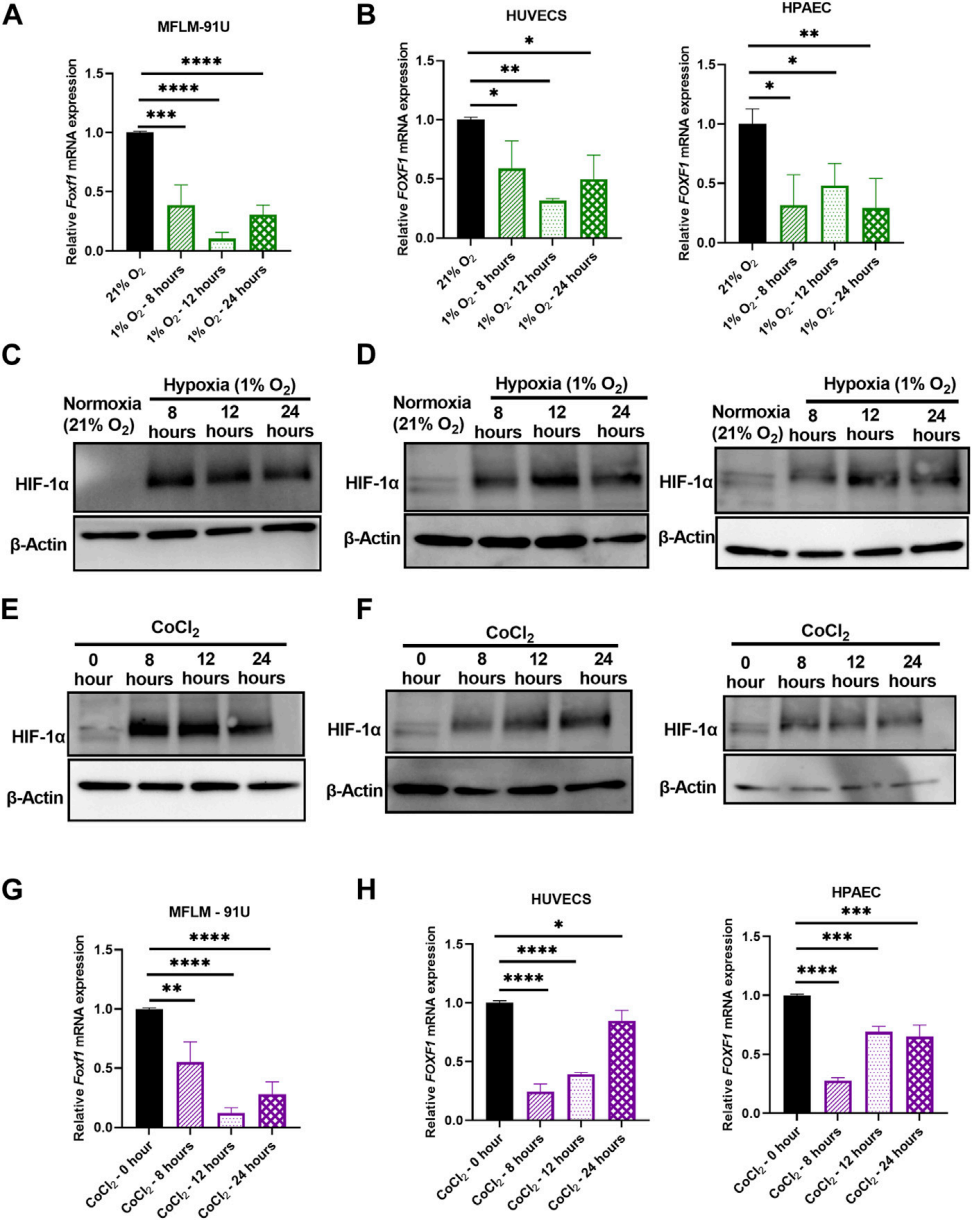
Hypoxia inhibits *Foxf1* in lung endothelial cells *in vitro*. **(A)** *Foxf1* mRNA expression was decreased in mouse endothelial MFLM 91U cells under hypoxic exposure (1% O_2_) for 8 hours, 12 hours, and 24 hours **(B)** *FOXF1* mRNA expression was decreased in human endothelial cells HUVEC (left) and HPAEC (right) under hypoxic exposure (1% O_2_) for 8 hours, 12 hours, and 24 hours **(C)** HIF-1α protein was induced in MFLM 91U cells under hypoxia (1% O_2_) from 8 hours to 24 hours **(D)** HIF-1α protein was induced in HUVEC (left) and HPAEC (right) under hypoxia (1% O_2_) from 8 hours to 24 hours **(E)** HIF-1α protein was induced in MFLM 91U cells upon treatment with 150 µM of cobalt (II) chloride for 8 hours, 12 hours, and 24 hours **(F)** HIF-1α was induced in HUVEC (left) and HPAEC (right) upon treatment with 150 µM of cobalt (II) chloride for 8 hours, 12 hours and 24 hours **(G)** *Foxf1* mRNA expression was decreased in MFLM 91U upon treatment with 150 µM of cobalt (II) chloride for 8 hours, 12 hours and 24 hours **(H)** *FOXF1* mRNA expression was decreased in HUVEC (left) and HPAEC (right) upon treatment with 150 µM of cobalt (II) chloride for 8 hours, 12 hours, and 24 hours. All qRT-PCR analyses employed β-Actin (*Actb*) as the housekeeping gene. Experiments were conducted three times. All values in this figure are shown as mean ± SD. *, *p* < 0.05; **, *p* < 0.01; ***, *p* < 0.001 from one-way ANOVA followed by Dunnett’s test.

Next, we tested if hypoxia directly inhibits *Foxf1 in vitro*. We performed time-course studies using 3 different endothelial cell lines, including embryonic mouse lung MFLM-91U endothelial cells, human umbilical vein endothelial cells (HUVECs) and human pulmonary artery endothelial cells (HPAECs). Cells were subjected to hypoxia (1% oxygen) for 8 hours, 12 hours, and 24 hours. Controls included cells exposed to normoxia (21% oxygen). *Foxf1* mRNA was measured using qRT-PCR. *Foxf1* mRNA was significantly decreased at 8 hours of hypoxia and remained downregulated until 24 hours in all 3 cell lines (Figures 3A, B). Hypoxia inducible factor 1-α (HIF-1α) is the master regulator of hypoxic responses. Under normoxic conditions, it is degraded via the ubiquitin-proteosome pathway, whereas under hypoxic conditions its degradation is inhibited and the HIF-1α protein is accumulated in the cell (Rhim et al., 2013; Vriend and Reiter, 2016) Therefore, we examined if the HIF-1α protein is increased at the time points at which we observed hypoxia-mediated downregulation of *Foxf1*. Western blot analysis for HIF-1α was performed in whole endothelial cell lysates at each time point after hypoxia. As seen in Figures 3C, D; Supplementary Figure S1A–C, HIF-1α protein was increased in all 3 endothelial cell lines at 8, 12, and 24 hours after the initiation of hypoxic treatment.

To further confirm our observations from the *in vitro* study of hypoxia, we utilized a chemical model of hypoxia. Cobalt (II) chloride is a widely used hypoxia mimetic (Muñoz-Sánchez and Chánez-Cárdenas, 2018). It is currently accepted that Co^2+^ substitutes Fe^2+^ in the prolyl hydroxylase (PHD) enzyme and inhibits it. Subsequently, proteasomal degradation of HIF-1α under normoxic conditions is inhibited, thus leading to its stabilization. To mimic the study in the hypoxia chamber, we treated endothelial MFLM-91U, HUVEC, and HPAEC cells with 150 µM of cobalt (II) chloride for 8, 12, and 24 hours to mimic hypoxia. Untreated cells were used as controls. To confirm if HIF-1α was increased after the cobalt (II) chloride treatment, we performed a Western blot analysis. HIF-1α protein expression was low in untreated cells, but it increased after 8, 12, and 24 hours after the cobalt (II) chloride treatment in all 3 endothelial cell lines (Figures 3E, F; Supplementary Figures 1D–F). Next, we measured *Foxf1* mRNA levels in these cells by qRT-PCR. MFLM-91U cells exhibited a maximum reduction in *Foxf1* mRNA at 12 hours under 1% oxygen (Figure 3A) and similar results were observed after the cobalt (II) chloride treatment (Figure 3G). HUVECs under 1% oxygen showed the maximum *Foxf1* reduction at 12 hours; however, under cobalt (II) chloride treatment the maximum reduction was observed at 8 hours but sustained until 12 hours (Figures 3B, H). HPAECs under 1% oxygen demonstrate comparable levels of *Foxf1* mRNA at all time points, while under cobalt (II) chloride treatment, the maximum reduction was observed at 8 hours (Figures 3B, H). Taken together, these data demonstrate that hypoxia directly inhibits *Foxf1* expression in endothelial cells *in vitro.*

### Hypoxia represses *Foxf1* expression in lung endothelial cells through HIF-1α

**FIGURE 4:**
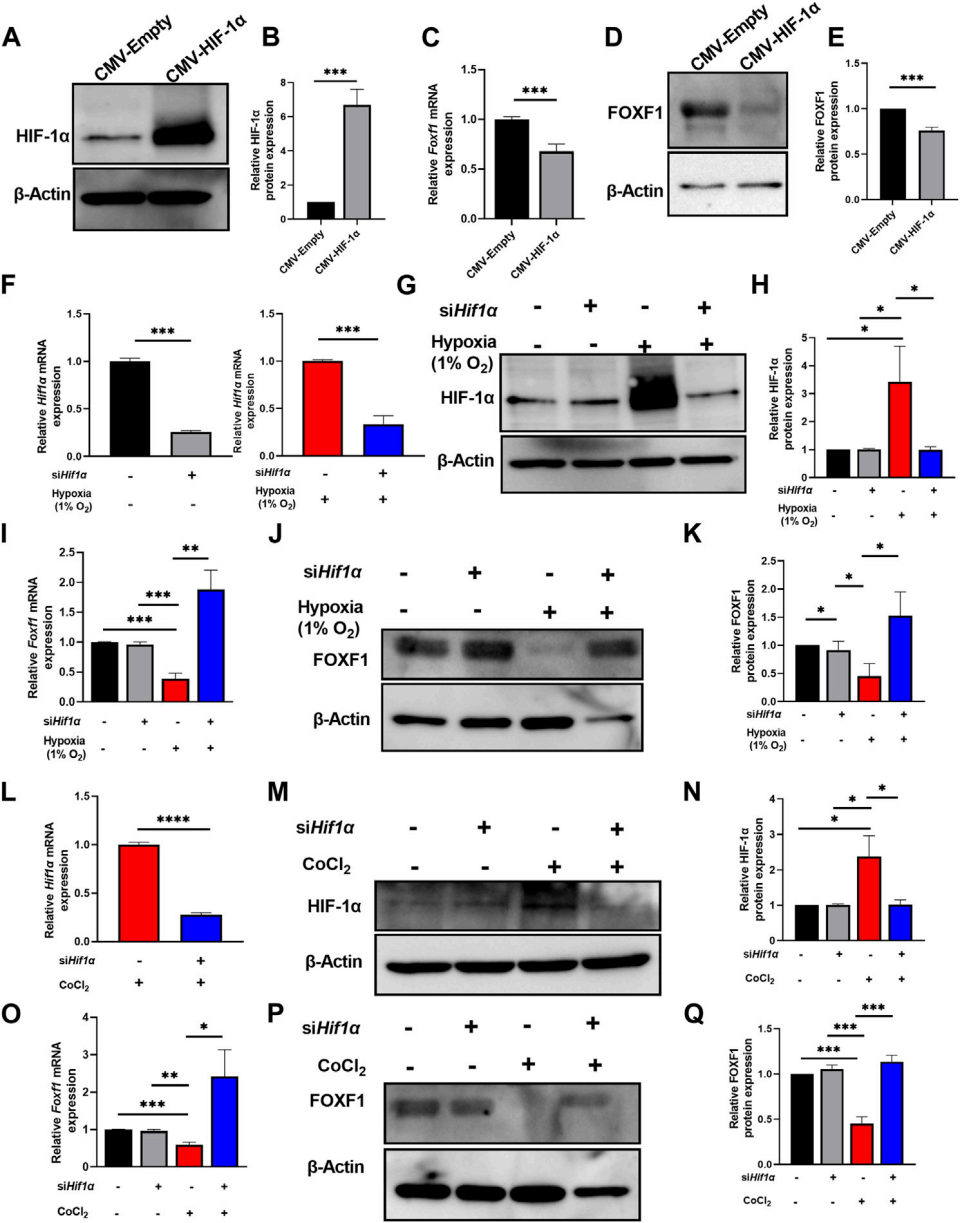
Hypoxia represses *Foxf1* expression in lung endothelial cells through HIF-1α. **(A,B)** HIF-1α protein was overexpressed in MFLM 91U cells transfected with CMV-HIF-1α under normoxic conditions. Protein expression is represented relative to control cells transfected with a CMV-empty vector. **(C)** *Foxf1* mRNA expression was decreased in MFLM-91U cells with overexpression of HIF-1α under normoxic conditions. **(D,E)** FOXF1 protein expression was decreased in MFLM-91U cells with overexpression of HIF-1α under normoxic conditions. Protein expression is represented relative to control cells transfected with a CMV-empty vector. **(F)** *Hif1α* was knocked down at an efficiency of 75% under normoxic (left) and 67% under hypoxic (right) conditions. MFLM-91U cells were transfected with non-targeting siRNA (siNT) or si*Hif1α* and subsequently exposed to normoxia (21% O_2_) or hypoxia (1% O_2_). **(G,H)** Knockdown of *Hif1α* prevents induction of HIF-1α protein under hypoxia (1% O_2_). MFLM-91U cells transfected with non-targeting siRNA (siNT) or si*Hif1α were* exposed to normoxia (21% O_2_) or hypoxia (1% O_2_) for 12 hours. Protein expression is represented relative to control cells transfected with non-targeting siRNA (siNT) in normoxia. **(I)** *Foxf1* mRNA expression is restored under hypoxic conditions upon knockdown of *Hif1α*. qRT-PCR was performed on MFLM-91U cells transfected with non-targeting siRNA (siNT) or si*Hif1α* and exposed to normoxia (21% O_2_) or hypoxia (1% O_2_) for 12 h **(J,K)** FOXF1 protein expression is restored under hypoxic conditions upon knockdown of *Hif1α.* Protein expression is represented relative to control cells transfected with non-targeting siRNA (siNT) in normoxia. **(L)** *Hif1α* was knocked down at an efficiency of 73% under cobalt (II) chloride treatment. MFLM-91U cells were transfected with non-targeting siRNA (siNT) or si*Hif1α* and subsequently treated with cobalt (II) chloride. **(M,N)** Knockdown of *Hif1α* prevents induction of HIF-1α protein under cobalt (II) chloride treatment. MFLM-91U cells were transfected with non-targeting siRNA (siNT) or si*Hif1α* followed by treatment with cobalt (II) chloride for 12 h. Protein expression is represented relative to control cells transfected with non-targeting siRNA (siNT) and no cobalt (II) chloride treatment. **(O)** *Foxf1* mRNA expression is restored under cobalt (II) chloride treatment upon knockdown of *Hif1α*. qRT-PCR was performed on MFLM-91U cells transfected with non-targeting siRNA (siNT) or si*Hif-1α* and subsequently treated with cobalt (II) chloride for 12 h **(P,Q)** FOXF1 protein expression is restored under cobalt (II) chloride treatment upon knockdown of *Hif1α*. Protein expression is represented relative to control cells transfected with non-targeting siRNA (siNT) and no cobalt (II) chloride treatment. All qRT-PCR analyses employed β-Actin (*Actb*) as the housekeeping gene. Western blot quantifications were performed using densitometric analysis on ImageJ software. Experiments were conducted three times. All values in this figure are shown as mean ± SD. *, *p* < 0.05; **, *p* < 0.01; ***, *p* < 0.001 from Student’s t test (two-tailed).

Next, we investigated the molecular mechanism whereby hypoxia inhibits *Foxf1* expression in endothelial cells. Observations from the *in vitro* and *in vivo* studies indicated a relationship between hypoxia, increased HIF-1α protein levels, and reduced *Foxf1* gene expression (Figures 1–3). Therefore, we tested if HIF-1α plays a role in the repression of endothelial *Foxf1* gene after hypoxia. First, we overexpressed HIF-1α in endothelial MFLM-91U cells under normoxic conditions using transient transfection with CMV-HIF-1α plasmid. Overexpression of HIF-1α led to the accumulation of HIF-1α protein (Figures 4A, B), which was associated with decreased FOXF1 mRNA and protein as determined by qRT-PCR and western blot respectively (Figures 4C–E). Thus, overexpression of HIF-1α was sufficient to inhibit *Foxf1* even under normoxic conditions in endothelial cell cultures.

Second, to determine if inhibition of HIF-1α can restore the FOXF1 expression, we depleted *Hif1α* in MFLM-91U cells using *Hif1α*-specific siRNA (si*Hif1α*). *Hif1α* knockdown efficiency was approximately 75% as shown by qRT-PCR (Figure 4F). After *Hif1α* knockdown, we exposed the cells to hypoxia (1% oxygen) for 12 hours. After harvesting the cells, we measured HIF-1α and FOXF1. HIF-1α protein and mRNA were decreased in *Hif1α*-deficient cells compared to cells transfected with non-targeting siRNA (control) (Figures 4F–H). Based on FOXF1 mRNA and protein expression measurements, there was no difference in FOXF1 expression between control and si*Hif1α-*treated cells grown under normoxic conditions (Figures 4I–K). In contrast, si*Hif1α-*treated cells grown under hypoxia showed an increase in FOXF1 mRNA and protein (Figures 4I–K). Next, we mimicked these experiments using cobalt (II) chloride instead of 1% oxygen. We observed a similar rescue of FOXF1 mRNA and protein in si*Hif1α-*transfected cells exposed to cobalt (II) chloride (Figures 4L–Q). Taken together, our results indicate that hypoxia inhibits *Foxf1* gene expression in endothelial cells, at least in part, through HIF-1α dependent mechanism.

## Discussion

Maintenance of endothelial barrier function is crucial for the maintenance of normal lung homeostasis and its repair following injury (Mehta and Malik, 2006). Pulmonary endothelial cells are important mediators in the exchange of gases, water and macromolecules between blood and alveolar tissue. They function as regulators of homeostasis and inflammation by secreting various cytokines and chemokines. Additionally, they regulate vascular tone and interact with other vascular cell types and inflammatory cells. Endothelial barrier function is known to be maintained by various factors such as angiopoietin-1 and sphingosine-1 phosphate (Jr et al., 1969; Gale and Yancopoulos, 1999). Cai *et al.* examined the function of transcription factor Forkhead box F1 (FOXF1) in quiescent endothelial cells of adult mouse lungs and identified it to be essential for the maintenance of lung homeostasis and prevention of edema after lung injury (Cai et al., 2016). They show that FOXF1 is a critical transcriptional regulator of endothelial cells and maintains endothelial barrier function by transcriptionally activating the *S1pr1* promoter. Additionally, using heterozygous Pdgfb-iCreER/Foxf1^+/−^ mice, they demonstrate that deletion of one allele of *Foxf1* was enough to increase the susceptibility of these mice to acute lung injury (ALI). In a recent study focusing on fibrosis-associated endothelial cells, our group found that *Foxf1* is one of the most downregulated genes in the bleomycin-injured mouse model of pulmonary fibrosis (Bian et al., 2023). Thus, restoring or increasing *Foxf1* levels in endothelial cells should be considered to alleviate pulmonary fibrosis. Additionally, *Foxf1* plays an anti-fibrotic role by preventing the transition of fibroblasts into myofibroblasts by inhibiting the CDH2-CDH11 cadherin switch (Black et al., 2018). Taken together, these studies indicate that *Foxf1* plays a crucial role in lung injury and repair. However, what causes the downregulation of endothelial *Foxf1* upon lung injury is still not well understood.

In the present study, we demonstrate that hypoxia plays a role in the downregulation of FOXF1 in endothelial cells both *in vivo* and *in vitro.* Using *in vitro* studies, we identified that this downregulation is at least in part through HIF-1α. In our previously published study by Bian *et al.*; we detected *Foxf1* expression in arterial, venous, aCap and gCap sub-clusters of normal lungs and its expression was decreased in all the sub-clusters upon bleomycin-induced lung injury. Therefore, it is possible that the HIF-1a/FOXF1 regulatory mechanism is active in several types of pulmonary endothelial cells. Consistent with this hypothesis, we observed similar effects in different cell lines with high FOXF1 expression levels: MFLM-91U (fetal microvascular endothelial cells), HUVECS (venous endothelial cells) and HPAECS (arterial endothelial cells).

Despite the lung being a well-oxygenated organ, hypoxia is a characteristic of several lung injuries that can be induced chemically or mechanically. Pathological conditions that cause hypoxia include pneumonia, smoke inhalation, lung cancer and influenza or other viruses such as SARS-CoV-2. Hypoxia is also a prominent feature of pulmonary fibrosis (Senavirathna et al., 2018) and patients with interstitial lung diseases and pulmonary fibrosis suffer from varying degrees of dyspnoea. Further, the progression of fibrotic disease correlates with increasing hypoxia (Flaherty et al., 2006).

Herein, we demonstrate that one dose of intratracheal instillation of bleomycin was sufficient to induce hypoxia in the lungs of mice 3 days post bleomycin injury. While induction of hypoxia upon bleomycin injury has been reported before (Akahori et al., 2022), this is the first study showing that it is induced within 3 days. Interestingly, day 3 was also the time point at which we have previously detected a decrease in *Foxf1* mRNA expression (Bian et al., 2023) using the same model of injury. Thus, indicating that hypoxia could be a microenvironmental factor responsible for the downregulation of endothelial *Foxf1* upon injury. Moreover, several studies have demonstrated the role of hypoxia in the development and progression of pulmonary fibrosis (Tzouvelekis et al., 2007; Mizuno et al., 2009; Weng et al., 2014; Choi et al., 2015; Senavirathna et al., 2018). Interestingly, exposure of mice to hypoxia only resulted in a decrease in endothelial FOXF1 expression compared to mice placed in room air. Exposure of mouse and human endothelial cell lines to a hypoxic environment or chemically induced hypoxia reiterated the observations *in vivo*. Intriguingly, analysis of the RNA seq dataset from the lungs of deceased patients who were hypoxemic also revealed a decreased expression of *FOXF1*. Taken together, all these data strengthened our observation that hypoxia initiates a downregulation in endothelial *FOXF1*.

Adjusting to altering oxygen availability is essential for the survival of all organisms. Hypoxia inducible factors (HIFs) are master regulators of response to hypoxia (Semenza, 2007; Semenza, 2011; Shimoda and Semenza, 2011; Jiang et al., 2019) and are known to be involved in disease pathogenesis (Semenza, 2007; Semenza, 2011). HIF-1α is vital for oxygen homeostasis and it shows a ubiquitous pattern of expression.

Interestingly, the present study reveals that *in vitro* downregulation of FOXF1 by hypoxia in endothelial cells occurs in HIF-1α dependent manner. Cobalt (II) chloride has been reported to chemically induce hypoxia *in vitro* by stabilizing HIF-1α (Dai et al., 2012). Utilizing the treatment of cells with cobalt (II) chloride and by knockdown and overexpression of HIF-1α, we demonstrated that FOXF1 is downregulated via HIF-1α. Although HIF-1α has been widely known as a transcriptional activator, it has recently been demonstrated to be both an activator as well as a repressor of gene expression (Manalo et al., 2005). Genes encoding Cystic Fibrosis Transmembrane Conductance Regulator (CFTR), and adenosine kinase (AK) in endothelial cells have been reported to be repressed via HIF-1α (Morote-Garcia et al., 2008; Zheng et al., 2009).

Several limitations are present in the current study. First, at present, we do not know the molecular mechanism by which HIF-1α represses endothelial FOXF1. The repression could be direct or indirect. HIF-1α has been previously reported to repress peroxisome proliferators–activated receptor alpha (*PPAR-α*) and the *ENT1* gene through hypoxia response element (HRE) present on the antisense strand (Narravula and Colgan, 2001; Eltzschig et al., 2005). This indicates potential directionality in transcriptional activity. However, Manalo *et al.* did a comparison of transcriptionally repressed genes by hypoxia and constitutively active HIF-1α and did not find any unique patterns (Manalo et al., 2005). Another potential mechanism could be that HIF-1α activates a transcriptional repressor, which then represses FOXF1. Thus, more work will be required to define the nature of HIF-1α mediated repression. Secondly, we do not know if HIF-1α represses the *Foxf1* gene *in vivo.* Endothelial-specific HIF-1α knockdown or delivering HIF1-α inhibitor to endothelial cells could be utilized to assess if HIF-1α inhibition can restore endothelial FOXF1 and subsequently rescue the phenotype of lung injury.

In summary, the present studies indicate that hypoxia plays a role in the repression of endothelial FOXF1 post-lung injury. HIF-1α knockdown and overexpression studies point towards the role of HIF-1α coordinating this response.

## Data Availability

Publicly available datasets were analyzed in this study. This data can be found here: https://www.ncbi.nlm.nih.gov/geo/query/acc.cgi?acc&equals;GSE117261. Gene Expression Omnibus (GEO) accession number GSE117261.

## Supplementary material

SUPPLEMENTARY FIGURE S1 Exposure of cells to hypoxia (1% oxygen) or cobalt (II) chloride induces HIF-1α protein expression. HIF-1α protein expression is induced under hypoxic exposure (1% O_2_) from 8 hours to 24 hours in mouse endothelial MFLM-91U cells **(B)** HUVECS **(C)** HPAECS. Protein expression is represented relative to control cells cultured under normoxia. HIF-1α protein expression is induced upon treatment with 150 µM cobalt (II) chloride from 8 hours to 24 hours in **(D)** mouse endothelial MFLM-91U cells **(E)** HUVECS **(F)** HPAECS. Protein expression is represented relative to control cells cultured under cobalt (II) chloride treatment for 0 hours. Experiments were conducted three times. Western blot quantifications were performed using densitometric analysis on ImageJ software. All values in this figure are shown as mean ± SD. *, *p* < 0.05; **, *p* < 0.01; ***, *p* < 0.001 from one-way ANOVA followed by Dunnett’s test.
