# Supplementary figures and images for: Hypoxia represses FOXF1 in lung endothelial cells through HIF-1α
Source: Front Physiol. 2024 Jan 11;14:1309155. doi: 10.3389/fphys.2023.1309155 (PMC10809398; doi:10.3389/fphys.2023.1309155)

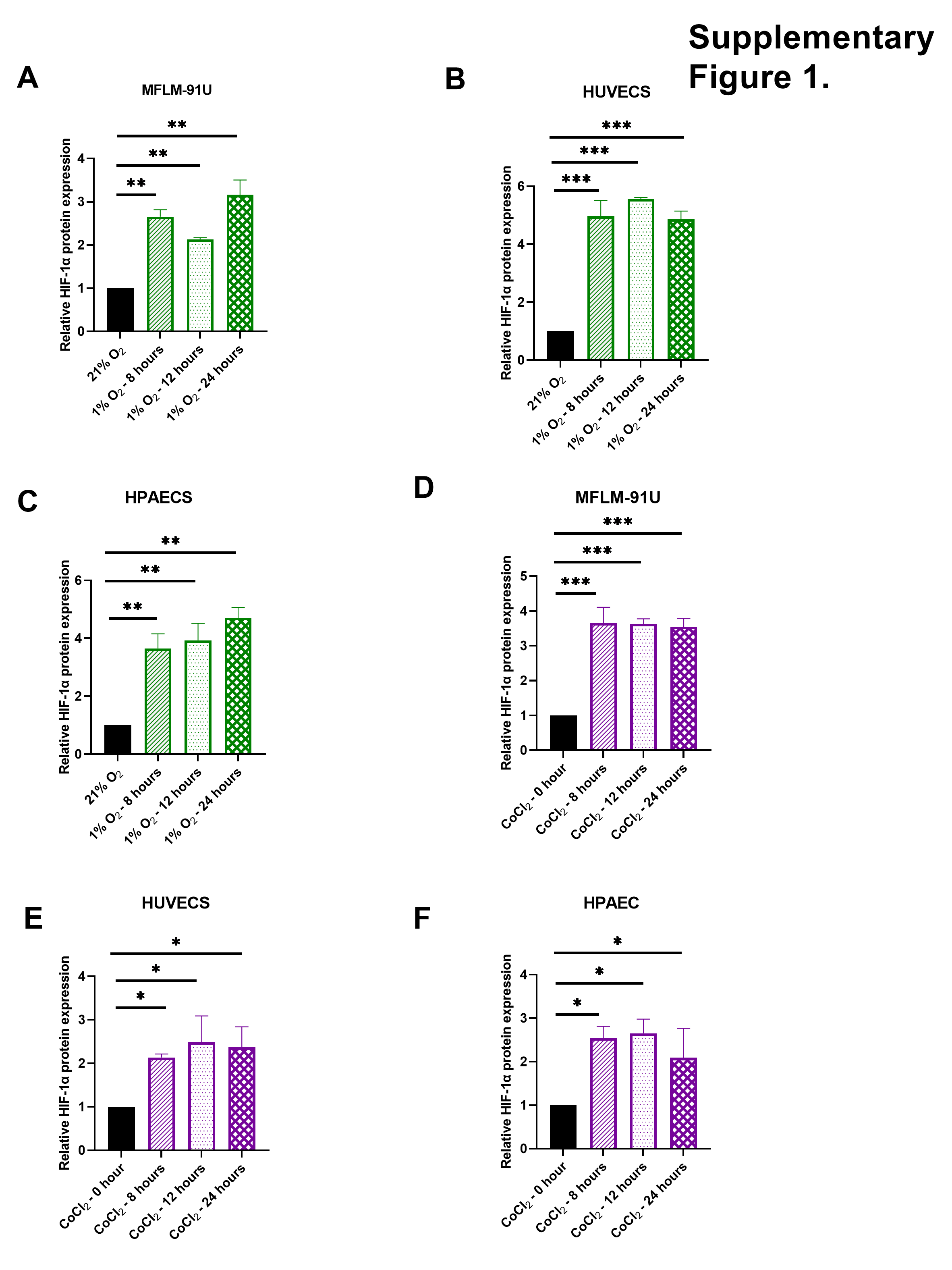

Supplement: Supplementary file 1 [file Image1.TIF]
